# Ki-67 expression and patients survival in lung cancer: systematic review of the literature with meta-analysis

**DOI:** 10.1038/sj.bjc.6602233

**Published:** 2004-11-16

**Authors:** B Martin, M Paesmans, C Mascaux, T Berghmans, P Lothaire, A-P Meert, J-J Lafitte, J-P Sculier

**Affiliations:** 1 Critical Care Department and Thoracic Oncology, Institut Jules Bordet, Centre des Tumeurs de l'Université Libre de Bruxelles, Brussels, Belgium; 2 Data Centre, Institut Jules Bordet, Centre des Tumeurs de l'Université Libre de Bruxelles, Brussels, Belgium; 3 Department of Surgery, Institut Jules Bordet, Centre des Tumeurs de l'Université Libre de Bruxelles, Brussels, Belgium; 4 FNRS (Fonds National de la Recherche Scientifique), Belgium; 5 Department of Chest Medicine and Thoracic Oncology, CHRU Lille, Hôpital Calmette, Lille, France

**Keywords:** Ki-67, proliferative factor, lung cancer, systematic review, meta-analysis

## Abstract

Among new biological markers that could become useful prognostic factors for lung carcinoma, Ki-67 is a nuclear protein involved in cell proliferation regulation. Some studies have suggested an association between Ki-67 and poor survival in lung cancer patients. In order to clarify this point, we have performed a systematic review of the literature, using the methodology already described by our Group, the European Lung Cancer Working Party. In total, 37 studies, including 3983 patients, were found to be eligible. In total, 49% of the patients were considered as having a tumour positive for the expression of Ki-67 according to the authors cutoff. In all, 29 of the studies dealt with non-small-cell lung carcinoma (NSCLC), one with small-cell carcinoma (SCLC), two with carcinoid tumours and five with any histology. In terms of survival results, Ki-67 was a bad prognosis factor for survival in 15 studies while it was not in 22. As there was no statistical difference in quality scores between the significant and nonsignificant studies evaluable for the meta-analysis, we were allowed to aggregate the survival results. The combined hazard ratio for NSCLC, calculated using a random-effects model was 1.56 (95% CI: 1.30–1.87), showing a worse survival when Ki-67 expression is increased. In conclusion, our meta-analysis shows that the expression of Ki-67 is a factor of poor prognosis for survival in NSCLC.

Molecular biology by the analysis and characterisation of proteins and genes involved in cancer development, could improve the knowledge of prognostic factors. By using the methodology of systematic reviews of the literature with meta-analyses, our Group has shown a prognostic role for several biological factors in lung cancer: p53 (Steels *et al*, 2001), Bcl-2 (Martin *et al*, 2003), vascular endothelial growth factor (VEGF) (Delmotte *et al*, 2002), epidermal growth factor receptor (EGFR) (Meert *et al*, 2002a), Neu (Meert *et al*, 2003) and microvessel density (Meert *et al*, 2002b).

Another field to explore by a meta-analysis is tumour proliferation, for example by the expression of Ki-67. The proliferation rate has been related to survival prediction (Murray and Kirschner, 1989; Pardee, 1989) and tumour cell kinetics studies have indicated a relationship between high cell proliferation rates and tumour aggressiveness (Tubiana and Courdi, 1989). Ki-67, a nonhistone protein, is a DNA-binding nuclear protein expressed throughout the cell cycle in proliferating cells, but not in quiescent (G_0_) cells. It has been used to distinguish growing from non growing cells (Seigneurin and Guillaud, 1991; Guinebretiere and Sabourin, 1997; Scholzen and Gerdes, 2000), although its exact function remains unclear.

Despite a large number of studies performed in lung cancer patients, the prognostic value of Ki-67 for survival remains controversial. Therefore, we performed a systematic review of the literature with meta-analysis to assess the prognostic value of its overexpression for survival.

## MATERIALS AND METHODS

### Publication selection

To be eligible for this systematic review, studies had to deal with lung cancer only, to evaluate the relationship between Ki-67 status and survival, to measure Ki-67 expression in primary tumour and to be published as a full paper in the English or French language literature. Abstracts were excluded because of insufficient data.

Articles were identified by an electronic search on Medline using the following keywords: ‘lung neoplasm’, ‘lung adenocarcinoma’, ‘NSCLC’, ‘SCLC’, ‘Ki-67’, ‘Ki67’, ‘MIB-1’, ‘proliferative index’, ‘proliferative activity’, ‘mitotic index’, ‘mitotic count’. The bibliographies reported in all the identified studies were used for completion of the studies search. When authors reported, in several publications, on the same patients population, only the most recent or complete study was included into the analysis, in order to avoid overlapping between cohorts. The search ended on December 2002.

### Methodological assessment

In order to assess the study methodology, eight investigators (six physicians, one biostatistician and one biologist) read each publication independently, and scored them using the European Lung Cancer Working Party (ELCWP) scale. This scoring system has been previously described (Steels *et al*, 2001). The scores proposed by each reader were compared and a consensus value for each item was reached. The participation of many readers warranted a correct interpretation of the articles.

The score evaluated various aspects of methodology, grouped into four main categories: the scientific design, the description of the laboratory methods used to identify the presence of Ki-67 (protein, DNA/RNA or antibodies against Ki-67), the generalisability of results and the analysis of the study data. Each category had a maximum score of 10 points, and the overall maximal theoretical score was 40 points. When an item was not applicable, its value was not taken into account in the total of the concerned category. The final scores were expressed as percentages, higher values reflecting better quality methodology.

Studies included in the systematic review were called ‘eligible’ and those providing sufficient data for the meta-analysis ‘evaluable’.

### Statistical methods

A study was considered as significant if the *P*-value for the statistical test comparing the survival distributions between the groups with and without Ki-67 expression, was <0.05. A study was classed as ‘positive’ when Ki-67 expression was identified as a good prognosis factor for survival in univariate analysis. A study was called ‘negative’ if the Ki-67 overexpression was associated with a significant detrimental effect on survival. Finally, a study was called ‘not significant’ if no difference between groups expressing or not Ki-67 was detected.

The association between the score measurements or between a score measurement treated as a continuous variable and another continuous variable was measured by the Spearman rank correlation coefficient. The comparison between score measurement according to the value of a discrete variable was made by nonparametric Mann–Whitney (for dichotomic variables) or Kruskal–Wallis (for nominal variables with multiple classes) tests.

For the quantitative aggregation of the survival results, the impact of Ki-67 expression on survival was measured by hazard ratio (HR). For each study, this HR was estimated by a method that depended on the results provided in the publication. The most accurate method was to retrieve the estimated HR and its standard error using two of the following parameters: the HR point estimate, the logrank statistic or its *P*-value, the O–E statistic (difference between numbers of observed and expected events) or its variance. If those data were not available, we looked for the total number of events, the number of patients at risk in each group and the logrank statistic or its *P*-value, allowing calculation of an approximation of the HR estimate. Finally, if the only useful data were in the form of graphical representations of the survival distributions, we extracted from them survival rates at specified times in order to reconstruct the HR estimate and its variance, with the assumption that the rate of patients censored was constant during the study follow-up (Parmar *et al*, 1998). Three independent persons read the curves to reduce the imprecision in the reading variations. If authors reported survival of three or more groups (for example, using several cutoff values for percentage of protein present in the nucleus), we pooled the results in order to make a comparison between two groups feasible.

The individual HR estimated were combined into an overall HR using the method published by Peto (Yusuf *et al*, 1985). We tested the homogeneity of the analysis by performing *χ*^2^ tests for heterogeneity. If the assumption of homogeneity had to be rejected, we used a random-effect model in place of a fixed-effect model. By convention, an observed HR>1 implied a worse survival for the group with positive Ki-67 expression. This impact of Ki-67 on survival was considered as statistically significant if the 95% confidence interval (CI) for the overall HR did not overlap 1.

For each study, the survival of the overall patients population was analysed, when available. If not, the HR was estimated on subgroups. If survival was reported separately for particular subgroups, these results were treated in the meta-analysis of the corresponding subgroups.

## RESULTS

### Studies characteristics

In total, 42 studies published between 1991 and 2002 were selected for this systematic review (Tungekar *et al*, 1991; Hayashi *et al*, 1993; Pence *et al*, 1993; Kawai *et al*, 1994; Costes *et al*, 1995; Giaccone *et al*, 1995; Harpole, Jr *et al*, 1995, 1996; Bohm *et al*, 1996; Fontanini *et al*, 1996; Giatromanolaki *et al*, 1996a, 1996b; Pujol *et al*, 1996; Bellotti *et al*, 1997; Ishida *et al*, 1997; Komaki *et al*, 1998; Mehdi *et al*, 1998; Soomro *et al*, 1998; D'Amico *et al*, 1999, 2000; Dazzi *et al*, 1999; Dingemans *et al*, 1999, 2001; Santinelli *et al*, 1999; Cagini *et al*, 2000; Carvalho *et al*, 2000; Demarchi *et al*, 2000; Hommura *et al*, 2000; Nguyen *et al*, 2000; Shiba *et al*, 2000, 2001; van de Vaart *et al*, 2000; Ferreira *et al*, 2001; Hayashi *et al*, 2001; Pelosi *et al*, 2001; Puglisi *et al*, 2001, 2002; Ramnath *et al*, 2001; Tsoli *et al*, 2001; Carbognani *et al*, 2002; Minami *et al*, 2002; Rigau *et al*, 2002). Five were excluded because an identical patients cohort was used in another selected publication (references excluded/included: D'Amico *et al*, 2000/1999; Giatromanolaki *et al*, 1996a/1996b; Harpole Jr. *et al*, 1995/1996; Puglisi *et al*, 2002/2001; Shiba *et al*, 2001/2000).

The main features of the 37 studies eligible for the systematic review are shown in Table 1

**Table 1:** Main characteristics and results of the eligible studies

			**NSCLC**											
	**All studies**		**Any stage (I–IV)**		**Locoregional (I–II)**		**Surgical treatment (I–III)**		**SCLC**		**Carcinoid tumours**		**Any histology any stage**	
	**Total**	**S**	**Total**	**S**	**Total**	**S**	**Total**	**S**	**Total**	**S**	**Total**	**S**	**Total**	**S**
Number of studies	37 (20)	15 (10)	10 (5)	4 (4)	8 (5)	3 (2)	11 (6)	3 (2)	1 (1)	1 (1)	2 (1)	2 (1)	5 (2)	2 (0)

NSCLC=non-small-cell lung cancer; SCLC=small-cell lung cancer; S=number of studies identifying Ki-67 positivity as a statistically significant prognostic factor; ()=number of studies evaluable for meta-analysis.

. A total of 29 studies dealt with NSCLC alone, while SCLC and carcinoid tumours were studied in 1 and 2 studies respectively. Five included tumours of any histology. Non-small-cell lung cancer studies included either all histological subtypes (*n*=25) or adenocarcinoma alone (*n*=4). Of the 29 NSCLC studies, 11 dealt with patients treated by surgery (stages I-IIIB); eight were performed in surgical stages (stages I–II), while 10 dealt with all stages (I–IV).

Immunohistochemistry (IHC) was the only technique used to detect the expression of Ki-67 protein. Various antibodies were used to assess Ki-67 expression: the clone MIB-1 in 68% of the studies and the Dako clone in 16%; the nature of the antibody was not described in 16%.

Of the 37 studies eligible for the systematic review, 17 provided insufficient data to perform a quantitative aggregation. The reasons for not including these studies in the meta-analysis were the following: no survival curve shown (*n*=2) (Bellotti *et al*, 1997; Carvalho *et al*, 2000); no *P*-value, HR or CI reported (*n*=8) (Kawai *et al*, 1994; Giatromanolaki *et al*, 1996a; Bellotti *et al*, 1997; Komaki *et al*, 1998; Santinelli *et al*, 1999; Hayashi *et al*, 2001; Ramnath *et al*, 2001; Carbognani *et al*, 2002); distribution of Ki-67 status not reported (*n*=11) (Tungekar *et al*, 1991; Kawai *et al*, 1994; Giaccone *et al*, 1995; Bellotti *et al*, 1997; Komaki *et al*, 1998; Soomro *et al*, 1998; Dazzi *et al*, 1999; Dingemans *et al*, 2001; Ferreira *et al*, 2001; Pelosi *et al*, 2001; Rigau *et al*, 2002).

### Studies results

As shown in Table 1, 15 of the 37 studies (40.5%). were negative showing Ki-67 expression as a bad prognostic factor for survival (10 of these 15 studies were evaluable for meta-analysis). The 22 remaining studies were not significant.

Out of the 29 studies performed in NSCLC, 10 (34.5%) were negative, including eight studies evaluable for meta-analysis. The studies related to SCLC and one of the two studies dealing with carcinoid tumours were negative.

A total of 3983 patients were included in the eligible studies. Ki-67 expression was clearly described for 2437 patients (61.2%): 49% (ranging from 10 to 78% according to the study) had a tumour with a positive immunostaining according to the authors cutoff (range 1–60%).

### Quality assessment

Before attempting to aggregate the results of the individual studies, a qualitative assessment of each study was performed. The global quality assessment score, ranged between 21.2 and 84.1%, with a median of 50.6% (Table 2A

**Table 2:** Methodological assessment by the ELCWP score, according to studies characteristics

	**Global score (%)**	**Design (/10)**	**Laboratory methodology (/10)**	**Generalisabilty (/10)**	**Results analysis (/10)**
*(A) All studies (n*=*37)*					
All studies	50.6	4.0	5	6.7	6.2
					
*Patient number*					
*r* Spearman	0.28	0.25	0.26	0.44	−0.005
*P*-value	0.09	0.13	0.12	**0.007**	0.97
					
*Date of publication*					
*r* Spearman	0.12	0.09	0.13	0.11	0.16
*P*-value	0.48	0.59	0.44	0.54	0.36
					
*Evaluable for the MA*					
Yes (*n*=20)	60.7	5.0	5.7	6.7	7.5
No (*n*=17)	45.8	3.0	4.3	5.8	5.0
*P*-value	**0.002**	**0.009**	**0.009**	0.57	**0.004**
					
*Significant results for survival*					
Yes (*n*=15)	52.5	4.0	5.0	6.7	6.2
No (*n*=22)	49.2	3.5	5.0	6.2	5.0
*P*-value	0.42	0.30	0.82	0.44	0.47
					
*(B) Studies evaluable for meta-analysis (n*=*20)*					
All studies	60.8	5.0	5.7	6.7	7.5
					
*Patient number*					0.59
*r* Spearman	0.45	0.31	0.27	0.57	0.80
*P*-value	**0.04**	0.18	0.24	**0.009**	
					
*Date of publication*					
*r* Spearman	0.20	−0.09	0.31	0.23	0.28
*P*-value	0.38	0.71	0.19	0.32	0.22
					
*Significant results for survival*					
Yes (*n*=10)	58.4	4.5	5.4	6.7	7.5
No (*n*=10)	68.0	5.5	6.1	6.2	6.9
*P-*value	0.54	0.34	0.40	0.88	0.57

Scale distributions are summarised by median value. MA=meta-analysis. The *P*-values in bold are significant.

). The design subscores had the lowest values. The most poorly described items (<30% of the maximum) were the *a priori* estimate of sample size required to conduct the study, the reproducibility control test between the experimenters, the initial disease work-up description and the number of unassessable samples with the reasons for their exclusion.

Weak and statistically nonsignificant correlations between the global score and either the number of patients included in the study (Spearman's correlation coefficient *r*=0.28, *P*=0.09) or the date of publication (Spearman's correlation coefficient *r*=0.12, *P*=0.48) were observed.

No statistically significant difference was found between the 15 significant and the 22 nonsignificant studies for the global score (median 52.5 in comparison to 49.2%, *P*=0.42), nor for the four subgroups scores.

A significant quality difference was observed between evaluable and nonevaluable studies for meta-analysis (median overall score: 60.7 *vs* 45.8%, *P*=0.002).

Scores of the 20 studies evaluable for the meta-analysis are reported in Table 2B. The overall quality score ranged between 21.2 and 84.1%, with a median of 60.8%. There was a significant correlation between the global score and the number of patients included in the study (Spearman's correlation coefficient *r*=0.45, *P*=0.04), with better scores in case of higher number of patients. The most poorly described items were similar to the all eligible studies. There was no significant difference between significant and nonsignificant studies for the global score with a median overall quality score of 58.4 and 68.0% respectively (*P*=0.54).

### Meta-analysis

The absence of any significant methodological qualitative difference between significant and nonsignificant studies led us to perform quantitative aggregations of the survival data of the 2233 patients included in the 20 studies. Only subgroup analyses were performed due to the heterogeneity of the studies: heterogeneity related to different histological types (NSCLC, SCLC or carcinoid), stages (localised, locoregional or extensive) or treatments. The subgroups analysed were defined according to histology and extent of the disease. The studies related to carcinoid tumours (*n*=1), small-cell-lung cancers (*n*=1) and the two evaluable studies related to any histology were not included in the present analysis, because their aggregation with other groups was not meaningful.

The hazard ratios were retrieved by one of the three methods reported in the Materials and Methods section. No study reported the summary statistics necessary to directly estimate the HR, but in four studies, the authors showed the individual data allowing us to make a direct estimation of the HR. In eight studies, the HR was approximated using the total number of events and the log-rank statistic or its *P*-value. For the eight remaining studies, the HR was extrapolated from the graphical representation of the estimated survival distributions.

**Figure 1:**
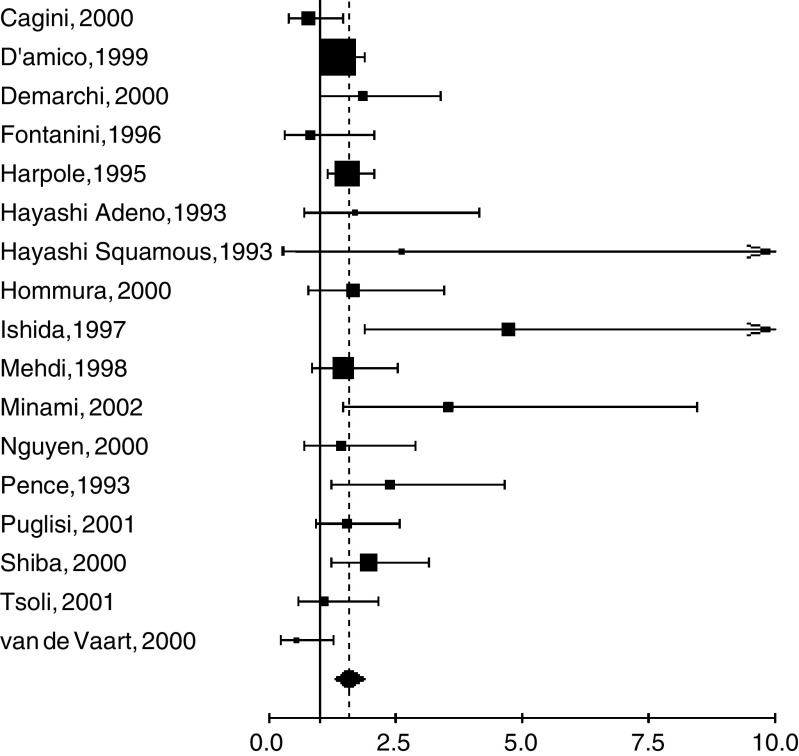
Results of the meta-analysis with all NSCLC studies. A hazard ratio (HR)>1 implies a worse survival for the group with no increased Ki-67 expression. The square size is proportional to the number of patients included in each study. The centre of the lozenge gives the combined HR for the meta-analysis and its extremities the 95% confidence interval.

The NSCLC subgroup included 16 studies with 1863 patients. One study (Hayashi *et al*, 1993) was divided into two subgroups. The aggregated survival data showed a poor survival prognosis in case of Ki-67 positivity (HR=1.55, 95% CI 1.34–1.78). There was no detectable heterogeneity between studies (*P*=0.12) but, as the χ^2^ test for heterogeneity was lacking from statistical power, we also used a random-effects model, with a result that was still statistically significant (HR 1.56, 95% CI 1.30–1.87) (Figure 1

).

We also analysed the data according to histology subtypes and tumour stage (Table 3

**Table 3:** HR value for NSCLC subgroups according to histology subtypes and stages

**Subgroup**	**Studies**	**Patients**	**Fixed effect HR (95% CI)**	**Heterogeneity test**	**Ramdom effect HR (95% CI)**
Adenocarcinoma	*n*=4	258	2.45 (1.66–3.64)	*P*=0.26	
Nonsquamous carcinoma	*n*=3	158	2.47 (1.32–4.57)	*P*=0.90	
					
Stage I	*n*=4	783	1.56 (1.26–1.93)	*P*=0.17	
Stage I–II	*n*=4	437	1.16 (0.82–1.66)	*P*=0.21	
Stage I–III	*n*=6	533	1.79 (1.40–2.28)	*P*=0.04	1.82 (1.26–2.64)

). For all the subgroups except stage I–II, the aggregation produced a statistically significant HR. The use of a random-effects model provided similar results.

## DISCUSSION

The present systematic review shows that the expression of Ki-67 in resected non-small-cell lung cancer is a poor prognostic factor for survival. We cannot extrapolate our results to metastatic NSCLC, to small cell lung cancer or to carcinoid tumours, because of a lack of adequate studies dealing with these specific presentations or histologies.

To perform the meta-analysis, we used the same methodology than in our previous systematic reviews (Meert *et al*, 1999, 2002a, 2002b; Mascaux *et al*, 2000; Sculier *et al*, 2001; Steels *et al*, 2001; Martin *et al*, 2003). The absence of statistically significant difference in quality scores between significant and nonsignificant publications allowed us to perform a quantitative aggregation of the individual studies results. Nevertheless, our approach does not eliminate all potential biases.

The review was restricted to articles published in English or French languages because other languages such as Japanese (Wang *et al*, 1997a, 1997b; Klein *et al*, 2000; Kayser *et al*, 2001; Sekine and Fujisawa, 2003) were not accessible to the readers. This selection could favour the positive studies that are more often published in English while the negative ones tend to be more often published in native languages (Egger *et al*, 1997). Moreover, our review took into account only fully published studies. We did not look for unpublished studies and abstracts because our methodology required data only in available in full publications order to perform methodology assessment and meta-analysis.

Meta-analysis based on individual data is considered by some authors as a gold standard (Stewart and Parmar, 1993). Systematic reviews of the literature should not be confused with meta-analyses of individual patients data. The first approach is based on fully published data and provides an exhaustive and critical analysis on the topic with an adequate methodology based on the criteria of Mulrow (1987). The second approach is, in fact, a new study taking into account all performed studies on the topic, published or not, requiring individual data updated by the investigators and is much more time-consuming. Nevertheless, our approach based on the literature provides similar results as shown by our meta-analysis on the role of prophylactic cerebral irradiation in small-cell lung cancer (Meert *et al*, 2001), where the results based on published data were the same as those found in the meta-analysis based on individual patients data (Auperin *et al*, 1999).

Another possible source of confusion is the use of the same cohort of patients in different publications. It might be difficult to avoid inclusion of some patients more than once in the meta-analysis, although publications clearly based on the analyses of the same patients cohorts were excluded. We have assumed that authors have been honest and have not reported the results from the same cohort of patients without mentioning it in their publications.

Studies assessing Ki-67 in lung cancer patient are heterogeneous. For reasons of homogeneity, our meta-analysis was performed only with studies dealing with NSCLC histology. By consequence, the Pujol study (Pujol *et al*, 1996) was not included because a few patients with SCLC were incorporated in the series. Nevertheless, similar results were obtained if this study was included but the heterogeneity increased. We restricted the analyses to the histological subtypes or tumour stages for which we had a sufficient number of studies. It was not possible, on the basis of published data, to adjust our results in a multivariate analysis although the usefulness of a new prognostic factor requires an independent prognostic value.

In total, 17 studies were excluded from the meta-analysis due to lack of the data necessary for aggregation. There is therefore a publication bias. These studies were not all statistically significant. It is known that nonsignificant studies are less frequently published or, if they are, with less detailed results, making them less assessable.

Another potential source of bias is related to the method for extrapolating the HR. If the authors did not report the individual HR together with its variance, we calculated it from the survival comparison statistic and its variance whenever possible. If not, we extrapolated it from the survival curves using several time points during follow-up for reading the corresponding survival rates, assuming that censored observations were uniformly distributed. This methodology is described in (Puglisi *et al*, 1998). Reading the survival rates on the graphical representation of the survival curves was performed independently by three of the authors, but this strategy does not eliminate completely inaccuracy in the extracted survival rates. Consequently, the estimated HR might be less reliable than when obtained from published statistics. Furthermore, we determined quite arbitrarily time intervals for reading survival rates on the curves, but we are not aware about any accepted methodology for the choice of the time intervals and their number. However, we compared our estimated HR and its statistical significance with the results published in each individual method and we did not identify any major contradiction between our results and the results available in the papers.

The techniques used to identify the expression of Ki-67 can also be a potential source of bias. Immunohistochemistry used to reveal Ki-67 protein was not always performed with the same antibody: clone Mib-1 in the majority of the studies (Costes *et al*, 1995; Bohm *et al*, 1996; Fontanini *et al*, 1996; Bellotti *et al*, 1997; Ishida *et al*, 1997; Komaki *et al*, 1998; Dazzi *et al*, 1999; Dingemans *et al*, 1999, 2001; Santinelli *et al*, 1999; Cagini *et al*, 2000; Demarchi *et al*, 2000; Hommura *et al*, 2000; Nguyen *et al*, 2000; Shiba *et al*, 2000, 2001; van de Vaart *et al*, 2000; Ferreira *et al*, 2001; Hayashi *et al*, 2001; Puglisi *et al*, 2001, 2002; Ramnath *et al*, 2001; Tsoli *et al*, 2001; Minami *et al*, 2002; Rigau *et al*, 2002), antibodies coming from a firm selling several clones but without mention of the clone used (Hayashi *et al*, 1993; Pence *et al*, 1993; Kawai *et al*, 1994; Giaccone *et al*, 1995; Pujol *et al*, 1996; Mehdi *et al*, 1998; Soomro *et al*, 1998; Carbognani *et al*, 2002); any mention of the antibodies used (Tungekar *et al*, 1991; Harpole Jr *et al*, 1995, 1996; Giatromanolaki *et al*, 1996a, 1996b; D'Amico *et al*, 1999, 2000; Carvalho *et al*, 2000;Pelosi *et al*, 2001). Two recent comparisons of four Ki-67 antibodies showed that there were considerable differences in the mean percentage of immunoreactive nuclei (Lindboe and Torp, 2002; Lindboe *et al*, 2003). According to these studies, the clone MIB-1 appears to have a higher sensitivity for detecting the Ki-67 antigen than other antibodies and it also gives the best visual staining. The antibodies concentration is also a factor having an influence on the staining result because of the intensity is correlated to the antibody concentration used. This aspect of the immunohistochemistry does not seem important for some authors because they do not specify the antibodies concentration (Tungekar *et al*, 1991; Hayashi *et al*, 1993; Costes *et al*, 1995; Harpole Jr *et al*, 1995, 1996; Giatromanolaki *et al*, 1996a, 1996b; Pujol *et al*, 1996; D'Amico *et al*, 1999, 2000; Dazzi *et al*, 1999; Santinelli *et al*, 1999; Nguyen *et al*, 2000; Shiba *et al*, 2000, 2001; Pelosi *et al*, 2001; Carbognani *et al*, 2002; Minami *et al*, 2002). Sometimes the immunohistochemical technique was performed without prior reaction for unmasked epitope on fixed tissue. Moreover, the cutoff defining a tumour with a Ki-67 positive staining is often arbitrary and varies according to the investigators, from a few percent to more than 50%. The use of different cutoff points for IHC is of critical importance, as shown by Lee *et al* (1995). Some investigators selected the cutoff point based on the minimum *P*-value approach, which can lead to seriously biased conclusions (Altman *et al*, 1994). If a chosen cutoff is often arbitrary, selection according to the median value of expression levels provides a more standardised approach to prognostic factors, although it may lead to some loss of information (Altman *et al*, 1994). In breast cancer, in a recent study, Spyratos *et al* (2002) compare Ki-67 scores with other classic factors measuring the proliferation rate, namely the mitotic index, which is a component of histologic grading system. They used five different Ki-67 cutoffs to define the most appropriate cutoff for distinguishing between tumours with low and high proliferation rates. These authors observed that with a Ki-67 cutoff of 10%, few tumours with low proliferation were misclassified. Conversely, a Ki-67 cutoff of 25% acceptably identified highly proliferative tumours. According to Spyratos *et al* (2002), the choice of cutoff depends on the clinical objective: if Ki-67 is used to exlude patients with slowly proliferating tumours from chemotherapeutic protocols, a cutoff of 10% will help to ovoid overtreatment. In contrast, if Ki-67 is used to identify patients sensitive to chemotherapy protocols, it is preferable to set the cutoff at 25%. The Ki-67 index should be combined with some other routinely used proliferative markers such as the mitotic index. In consequence, an optimal threshold still needs to be defined for Ki-67 and validate for lung cancer.

Cell division kinetics is an important predictor of the clinical outcome of various carcinoma. Cellular proliferation can be measured using a variety of methods. Mitotic indices have been widely used as part of various tumour grading methods (Elston and Ellis, 1993; Silverberg, 2000; Hilska *et al*, 2002). The mitotic index is a rapid an cost-effective tool for estimating tumour cell proliferation, and reasonable reproducibility can be achieved with a strictly standardized methodology (Elston and Ellis, 1993). Immunohistochemical determination of proliferation indices is an expanding area of research, based on detection of antigens presents during cell proliferation (Gerdes *et al*, 1991; Scholzen and Gerdes, 2000) such as Ki-67 with clone Mib-1. For many authors (Rudolph *et al*, 1998; Lehr *et al*, 1999; Spyratos *et al*, 2002), high Ki-67 clone Mib-1 was associated with a high mitotic index. Whatever the cutoff used for Ki-67 clone Mib-1, the mitotic indices were always the most discriminant variable. In clinical trials, Ki-67 clone Mib-1 could be used in conjunction with the mitotic index to ensure correct tumour classification on the basis of proliferative potential.

A few studies suggest a predictive role for Ki-67, in that an individual patient's tumour might be treated in a specific way based on its degree of Ki-67 expression. Tumour cell proliferation after hormonal treatment is a prognostic factor of recurrence in prostate cancer treated with neoadjuvant luteinizing hormone-releasing hormone treatment and radical prostatectomy (Ahlgren *et al*, 1999). The same results were obtained with patient treated by external beam radiotherapy (Khoo *et al*, 1999). Highly proliferative breast tumours are associated with shorter patient survival. Studies have suggested that highly proliferative tumours show increased sensitivity to neoadjuvant (Remvikos *et al*, 1989) and adjuvant chemotherapy (Simpson *et al*, 2000), regardless of the impact of such treatment on patient survival. In contrast, the rationale of chemotherapy for slowly proliferating tumours is controversial (Wenger and Clark, 1998).

Information on cell proliferation may be a useful adjunct to histologically based tumour classification in the understanding of tumour behaviour. In a variety of malignant neoplasms, significant correlations have been found between proliferative activity and metastatic potential, recurrence or overall prognosis (Hall and Levison, 1990). In our systematic review with meta-analysis, patients with Ki-67 positive tumours had shorter survival than those with Ki-67 negative tumours. The mechanism underlying the effect of Ki-67 protein expression on tumour progression as prognosis remains essentially uncertain. However, it has to be considered that positivity for the Ki-67 antigen may reflect the ability of a cell to continue to proliferate after the time of tumour resection. In fact, a cell would be positive as long as it is going to divide but the term going to divide refers not to the actual state of the cell but to an event in the future. The cell must make the final decision whether to divide at some time point during the cell cycle. In practice, these considerations should not be regarded as drawbacks. As the Ki-67 index (percentage of cells stained positive for the Ki-67 antigen) is directly based on a physiological parameter involved in cell proliferation, it may give an even better insight into the growth characteristics of a tumour, its susceptibility to certain drugs, and to the outcome of a patient that the estimation of the growth fraction. It is also evident that estimating the growth fraction alone is not sufficient to describe the tumour growth. The growth fraction (and the Ki-67 labelling index) relates only to the number (or fraction) of proliferative cells but not to the time needed for the completion of an intermitotic cycle. In other words, the estimation of the growth fraction gives information only about the state but not about the rate of proliferation; therefore, an additional marker would be helpful to assess this parameter. In the future, multiparameter analysis may provide a better means for analysing cell proliferation and tumour growth. This may not only improve the prognostic value but also be a prerequisite for choosing the appropriate type of therapy for each individual case.

In conclusion, our systematic review of the literature shows that expression of Ki-67 in patients with stages I–III NSCLC is a poor prognostic factor for survival. Our results were based on the aggregation of data obtained in univariate survival analyses performed in retrospective studies. In order to become a useful prognostic factor in the clinical practice, a standardisation of the immunohistochemistry technique is needed, particularly concerning the positivity threshold. In addition, the present results need to be confirmed by an adequately designed prospective study with an appropriate multivariate analysis taking into account the classical well-defined prognostic factors for survival in lung cancer patients.
